# Active temporal multiplexing of indistinguishable heralded single photons

**DOI:** 10.1038/ncomms10853

**Published:** 2016-03-21

**Authors:** C. Xiong, X. Zhang, Z. Liu, M. J. Collins, A. Mahendra, L. G. Helt, M. J. Steel, D. -Y. Choi, C. J. Chae, P. H. W. Leong, B. J. Eggleton

**Affiliations:** 1 Centre for Ultrahigh bandwidth Devices for Optical Systems (CUDOS), Institute of Photonics and Optical Science (IPOS), School of Physics, University of Sydney, New South Wales 2006, Australia; 2 School of Electrical and Information Engineering, University of Sydney, New South Wales 2006, Australia; 3 Department of Communication Engineering, School of Information Engineering, Guangdong University of Technology, 510006 Guangdong, China; 4 CUDOS and MQ Photonics Research Centre, Department of Physics and Astronomy, Macquarie University, New South Wales 2109, Australia; 5 CUDOS, Laser Physics Centre, Australian National University, Canberra, Australian Capital Territory 2601, Australia; 6 Department of Electrical and Electronic Engineering, University of Melbourne, Parkville, Victoria 3010, Australia

## Abstract

It is a fundamental challenge in quantum optics to deterministically generate indistinguishable single photons through non-deterministic nonlinear optical processes, due to the intrinsic coupling of single- and multi-photon-generation probabilities in these processes. Actively multiplexing photons generated in many temporal modes can decouple these probabilities, but key issues are to minimize resource requirements to allow scalability, and to ensure indistinguishability of the generated photons. Here we demonstrate the multiplexing of photons from four temporal modes solely using fibre-integrated optics and off-the-shelf electronic components. We show a 100% enhancement to the single-photon output probability without introducing additional multi-photon noise. Photon indistinguishability is confirmed by a fourfold Hong–Ou–Mandel quantum interference with a 91±16% visibility after subtracting multi-photon noise due to high pump power. Our demonstration paves the way for scalable multiplexing of many non-deterministic photon sources to a single near-deterministic source, which will be of benefit to future quantum photonic technologies.

## Abstract (web summary)

Deterministically generated single photons are useful for quantum communications, but the processes that create such light are often non-deterministic. Here, the authors enhance the single-photon output probability by multiplexing photons from four temporal modes using fibre-integrated optics.

## Graphical abstract

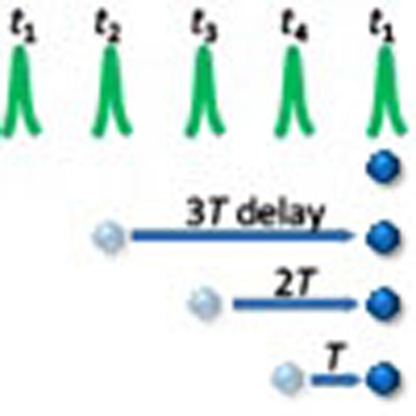

Single particles of light—photons—are a vital resource for the implementation of quantum-enhanced technologies such as optical quantum computing^1^ and simulation^2^. To make such technologies practical requires ideal single-photon sources, which can emit single photons on-demand and indistinguishable in all relevant degrees of freedom: central frequency, bandwidth, spatial mode, and polarization^3^,^4^. Despite recent progress on relaxing these requirements^5^,^6^, sources that meet the required thresholds do not yet exist. Two strategies have been proposed to develop the desired photon sources^7^. One is to use ‘single-emitter' quantum systems^8^,^9^,^10^,^11^ such as quantum dots or colour centres in diamond. These systems typically emit single photons nearly on-demand, with a recent demonstration showing that the emitted photons from a single quantum dot can be highly indistinguishable^12^. However, producing highly indistinguishable photons from distinct emitters remains challenging because of the difficulty of fabricating identical emitters at the nanoscale^13^,^14^. The alternative approach is to generate correlated photon pairs via spontaneous nonlinear optical processes, such as parametric down conversion or four-wave mixing in suitable crystals or waveguides, where the detection of one photon in a pair ‘heralds' the existence of its partner^15^,^16^,^17^. However the photon pair generation events are unpredictable (being associated with vacuum fluctuations) and contain contributions from multi-pair events. Indeed the probabilities of single- (*P*_1_) and multi-pair (*P*_>1_) events are both related to the mean number of pairs created per pump pulse *μ*. They both increase with *μ*, and *P*_>1_ increases more rapidly (to leading order it grows quadratically rather than linearly). Therefore, these sources are usually operated in the *μ*<<1 (and thus *P*_1_<<1) regime to minimize the multi-photon noise. Unfortunately, most useful quantum protocols require many simultaneous single-photon inputs in different modes, and as the success rate falls as (*P*_1_)^*n*^ for *n* input modes, operation quickly becomes impractical^4^,^5^. This has limited the world record quantum photonic demonstration to the eight-photon level^18^.

A promising solution is to actively multiplex non-deterministic photons in different spatial or temporal modes to enhance the probability of single-photon output^19^,^20^,^21^,^22^,^23^. Spatial multiplexing has been implemented in a few architectures^24^,^25^, but scaling quickly becomes infeasible as the number of photon sources and heralding detectors increases rapidly with the number of modes to be multiplexed^25^,^26^. Temporal multiplexing, proposed in refs 21, 22, 23, reuses the same detectors and photon-generation components, and thus is significantly more resource efficient and scalable. The scheme in ref. 23 requires an electronic circuit to extract timing information from the heralding photons, which is subsequently used to control a switching network that actively routes the heralded photons into a pre-defined temporal mode. Recently two groups have demonstrated initial experimental implementations of active temporal multiplexing^27^,^28^, but the remaining challenges are: managing the photons' arrival time to the accuracy of several picoseconds, and controlling their polarization to maintain the photons' indistinguishability; and developing ultra-low-loss integrated optical components so that the desired enhancement can be achieved in a scalable manner.

In the following, we aim to experimentally overcome all of these challenges using all-fibre-integrated low-loss optical devices and off-the-shelf fast electronic components, and to reveal the potential of this scheme for deterministic indistinguishable single-photon generation. We show a substantial increase in the heralded single-photon output probability at a given clock cycle with no concomitant increase in the multi-pair contamination.

## Results

### Temporal multiplexing scheme

**Figure 1:**
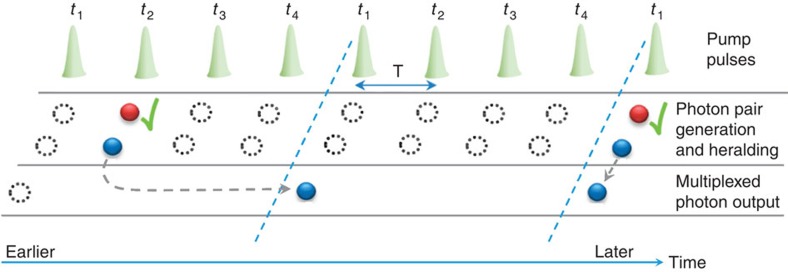
The principle of active temporal multiplexing. A nonlinear device is pumped by pulses separated in time by period *T*, each generating correlated photon pairs randomly. The two photons from each pair are spatially separated by frequency (colour) and the heralding photons (red) are detected, indicating the existence of the heralded photons (blue). Depending on the time bin in which a pair is generated, an appropriate delay is applied to the heralded photon so that it always appears in time bin *t*_1_ with a nominal period *NT* (*N*=4 in this work).

The principle of our demonstration is illustrated in Fig. 1. Compared with one pump pulse at period 4*T*, a group of pump pulses at period *T* are approximately four times as likely to generate a pair in the given time frame of 4*T* if the individual pulse energy is the same. However, the random nature of the generation process within each time bin remains the same. The situation changes after the heralded photons are actively delayed to time bin *t*_1_: if the switching network has sufficiently low losses, the heralded single-photon output probability at the 4*T* clock period will be increased.

### Optics and FPGA configuration

**Figure 2:**
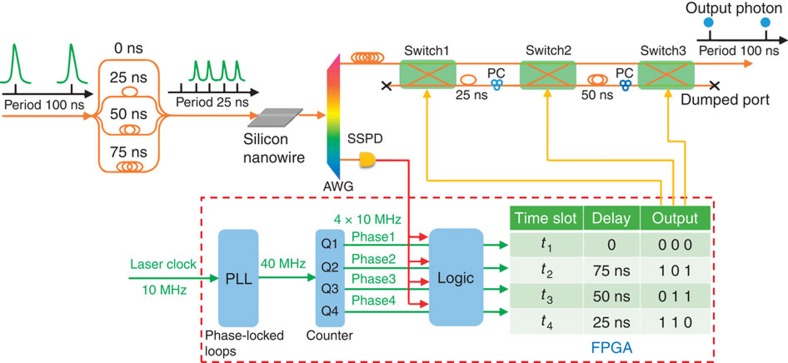
Experimental setup of four temporal mode multiplexing. Pulses from a mode-locked picosecond fibre laser are split to four copies using fibre couplers and tunable delay lines, and pump a silicon nanowire for spontaneous four-wave mixing. The 0, 25, 50 and 75 ns delays are all relative to the uppermost optical path. After pump blocking, frequency selection and spatial separation of the two photons of each pair, the heralding signals are analysed by a FPGA and the heralded photons are buffered using a long fibre delay to wait for the electronic decisions. The loss of the 200 m long buffer fibre is <0.1 dB. The FPGA configures the switching network to route the heralded photons into a single spatial-temporal mode. Logic ‘0' means the photon remains in the input (‘bar') channel; a ‘1' means the photon is routed to the cross channel.

To implement the scheme shown in Fig. 1, we design an experiment as shown in Fig. 2 (see Supplementary Fig. 1 and Supplementary Note 1 for the full setup). A mode-locked fibre laser with a repetition rate of 10 MHz (100 ns period) produces 10 ps pulses at 1,550 nm. Each pulse is split into four pulses spaced by 25 ns using two one-to-four fibre couplers and three tunable optical fibre delay lines. The four pulses then propagate along a 3-mm long nonlinear silicon nanowire, probabilistically generating correlated photon pairs via spontaneous four-wave mixing in the four time-bins^29^. As a result of energy conservation and phase matching, photon pairs are generated at frequencies symmetrically around the pump over a 6 THz bandwidth^29^. An arrayed waveguide grating (AWG, 100 GHz channel spacing and 50 GHz channel bandwidth) is used to select the photon pairs generated at 1,545 and 1,555 nm, block the pump, and spatially separate the two photons of each pair. The 1,555 nm photons are detected by a fast and low-noise niobium nitride superconducting single-photon detector as heralding signals. These signals contain the timing information of the 1,545 nm photons and are sent to a field-programmable gate array (FPGA) for analysis.

A phase-locked loop in the FPGA is used to lock to and multiply the laser's original 10 MHz clock to a 40 MHz clock. A finite state machine operating on the 40 MHz clock generates four non-overlapping clocks at four phases relative to the 10 MHz clock. A heralding photon detection signal from the superconducting single-photon detector is ANDed with each clock phase and an appropriate three binary-digit output latches. The output is connected to the switching network, so that the 1,545 nm photons are routed into the appropriate sequence of delay lines. All of these operations require the careful alignment of the clock with the optical pulses that contain the generated photons. This is done by optimizing the counts in a series of coincidence measurements, adjusting the tunable optical delay lines and tunable digital delays (see Supplementary Note 2).

### Loss management

To receive any benefit from a four temporal mode multiplexing setup, the switching network must have a total loss below the four times (that is, 6 dB) maximum expected enhancement. We use optical ceramic switches, made from ultra-low-loss lead lanthanum zirconium titanate^30^. These switches are fibre pigtailed and spliced with the fibre delay lines to minimize the loss of each path to around 2.8 dB, with ±0.3 dB difference between different routes (see Supplementary Fig. 2). Since this loss difference is much less than the overall loss in the experiment, its effect on output photon statistics is negligible. The setup described so far ensures indistinguishability in the spectral and temporal degrees of freedom, but we also require indistinguishability in polarization. In Fig. 2, the heralded photons from different time bins have the same polarization before they enter the switching network. However, they experience different optical paths to obtain the correct delays, and to minimize losses these components are not polarization maintaining. This is addressed using two polarization controllers applied to the two optical delay lines (see Supplementary Note 3). The additional loss introduced by each polarization controller is ∼0.1 dB.

### Multiplexing enhancement

**Figure 3:**
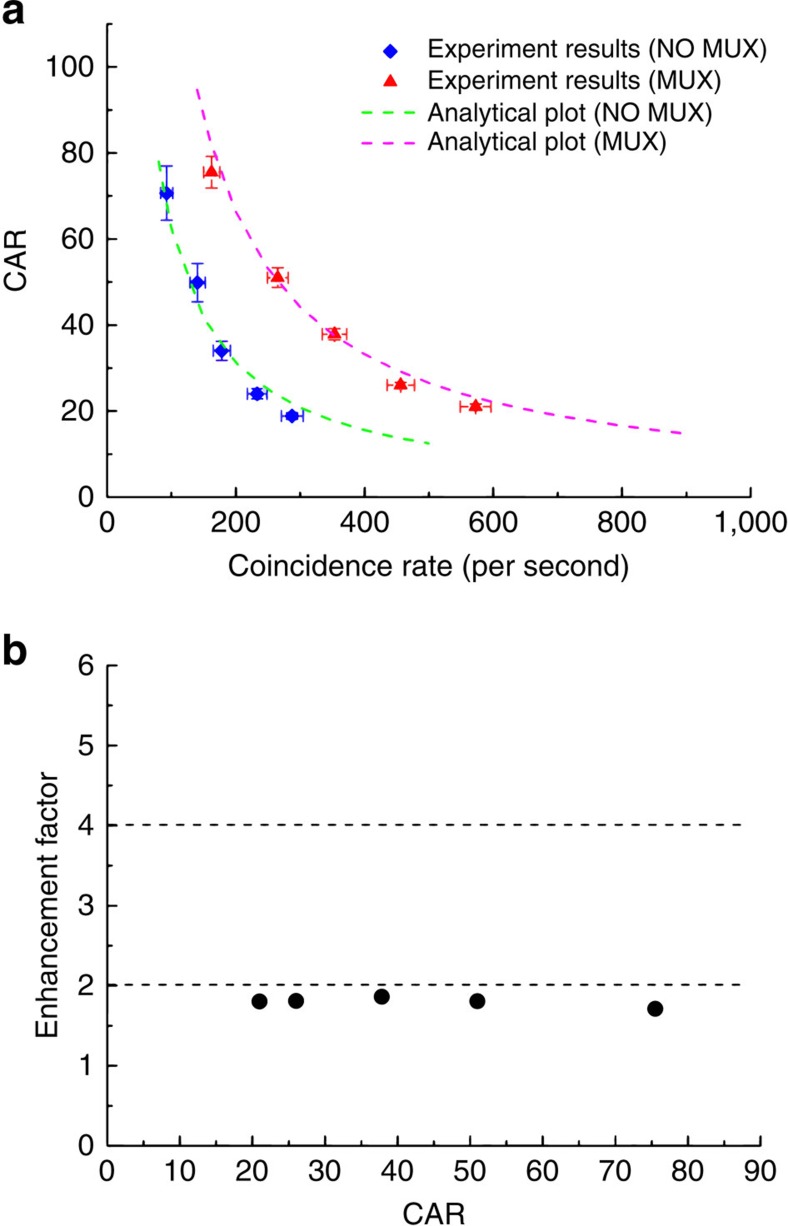
Comparison between sources with and without multiplexing. (**a**) CAR as a function of coincidence rates. Poisson error bars are used for the plots. Dashed lines are analytic plots using the model in ref. 25. (**b**) The inferred enhancement factors to the heralded single-photon output probability at each CAR level.

The key to verifying our design is to compare the heralded single-photon output probability per 100 ns clock period (that is, the original 10 MHz clock) at the same multi-photon noise level for sources with and without the multiplexing switching network. These two quantities are characterized by coincidence-to-accidental ratio (CAR) measurements^31^. When a pair of photons generated in the same pump pulse are detected and the detection signals sent to a time interval analyser, a coincidence is recorded. When photons generated from different pulses are detected, the coincidence represents an accidental coincidence. All of these coincidences (*C*) and accidentals (*A*) are recorded as a histogram by the time interval analyser (see Supplementary Fig. 1 and Supplementary Note 1), and CAR=*C*/*A*. The measured CAR as a function of the coincidence rate without multiplexing (NO MUX, that is, pumping at 10 MHz) is plotted in Fig. 3a, indicated by diamonds. The CAR decreases with the increased coincidence rate due to multi-pair noise and this is a typical feature of such measurements^25^,^29^,^31^. For comparison, we perform measurements at the same pump peak powers for the multiplexed source (MUX, that is, pumping at 40 MHz and adding the switching network to the setup). The results are plotted as triangles in Fig. 3a. The CAR still decreases with the increased coincidence rate because the original NO MUX sources have this feature. However, when compared with the NO MUX source, at the same CAR, that is, the same multi-pair noise level, the coincidence rates are nearly doubled. At the highest power level in our experiment, the detected coincidence rate has been increased from ∼300 s^−1^ for the source without multiplexing to nearly 600 s^−1^ after performing multiplexing. As simply doubling the number of pump pulses per period (that is, keeping the same peak power and without an active switching circuit) can lead to similar results in Fig. 3a (ref. 29), we express the improvement as an enhancement factor of MUX/NO MUX heralded single-photon output probability per 100 ns at the same CAR level. The enhancement is due to the fact that in the NO MUX case, there is a single pump pulse per 100 ns, while in the MUX case, there are four pump pulses per 100 ns; and the ratio between single- and multi-pair probabilities remains the same when the pulses have the same peak power. Taking into account the losses of waveguide-fibre coupling, spectral filters and the efficiency of detectors, we estimate the mean number of pairs per 100 ns clock period from the measured coincidence rate at each CAR level, and then infer the heralded single-photon output probabilities using a thermal distribution function for both NO MUX and MUX sources (see Supplementary Note 4). The enhancement factor at each CAR level is plotted in Fig. 3b as circles, showing that our four temporal mode multiplexing nearly enhances the heralded single-photon output probability by 100% (that is, 3 dB). The enhancement is less than the ideal factor of 4 (that is, 6 dB) because of the 3 dB loss of the switching network.

### Photon indistinguishability check

For the multiplexed source to be useful, the multiplexed heralded photons must be indistinguishable. This is tested by Hong–Ou–Mandel (HOM) quantum interference^32^. We build another heralded single-photon source based on a second 3-mm long silicon nanowire pumped by the same 10 MHz laser, but without multiplexing (see Supplementary Note 5). The photons from this second source are in a certain spatial-temporal state, that is, in an identical polarization state and at the accurate 100 ns clock cycle of the laser, and so they provide a reference to check if the multiplexed photons are indistinguishable. Note that as the AWG channels used to filter the generated photons have a slightly larger bandwidth (50 GHz) than the pump (10 ps transform limited pulses corresponding to 44 GHz), the photons to be interfered have some chance to be in different spectral modes. This may slightly reduce the HOM interference visibility^33^.

**Figure 4:**
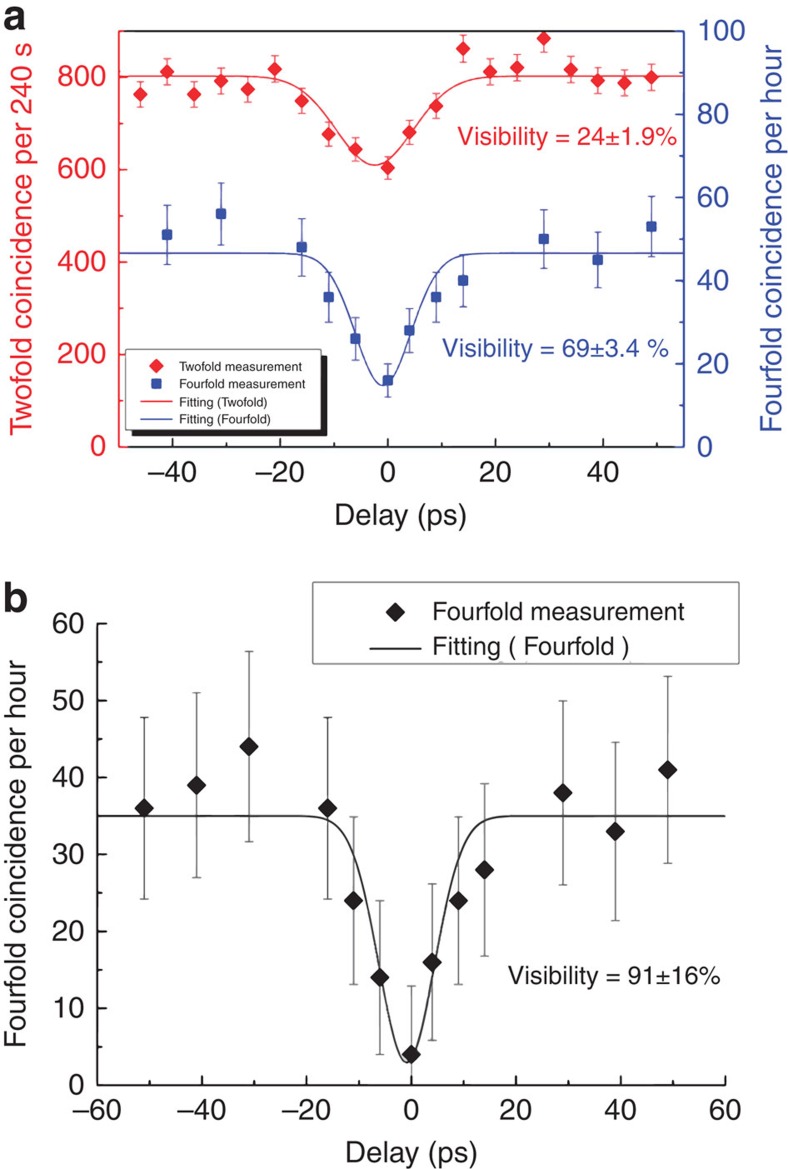
Indistinguishability measurement of the multiplexed photons. (**a**) Raw visibility of twofold (diamonds, left axis) and fourfold (squares, right axis) measurements. (**b**) Fourfold HOM dip visibility after subtracting multi-photon noise. Poisson error bars are used for the plots. Solid lines are Gaussian fits according to the spectral filtering shape in the experiment.

As the photons to be interfered at a 50:50 beam splitter must be heralded by their corresponding partner photons, the HOM interference here actually involves fourfold (or four photon) coincidence measurements^34^,^35^ (Supplementary Fig. 1). Because the fourfold coincidence rate from two separate sources is very low due to the low photon collection efficiency, we first perform a standard twofold interference measurement (that is, without heralding) to find the appropriate delay between photons from the two sources^34^. In this measurement, the pump powers are set at a level of CAR=18 for both. The twofold dip shows a raw visibility of 24±1.9% (diamonds in Fig. 4a). Then we take the fourfold HOM interference measurement, but at higher pump powers for both sources in order to have sufficient coincidence counts to make the statistics meaningful in a reasonable amount of measurement time (for example, 50 coincidences in 1 h) with our low-efficiency detectors. The cost is that the CAR drops to 7, and more multi-photon noise is generated and reduces the visibility of the HOM dip. We observe a fourfold HOM dip with raw visibility of 69±3.4% (squares in Fig. 4a), indicating that non-classical interference occurred between the multiplexed photons and the photons from the second source. To check that the residual photon distinguishability is not because of multiplexing but due to multi-photon noise at high pump powers, we measure the detector dark count and multi-photon contribution from each source (see Supplementary Note 5)^35^. Using these data we correct the raw data, which yields a visibility of 91±16% (Fig. 4b), clearly showing that the multiplexed photons are highly indistinguishable. This non-100% visibility is partly because of the large error bars resulting from the low count rates, and partly due to the photons' spectral distinguishability introduced by the slightly broader band filtering of the photons mentioned earlier.

## Discussion

Varnava *et al*.^5^ have shown that if the product of the detector efficiency with the source efficiency is greater than 2/3, then efficient linear optical quantum computation is possible. The detector efficiency has been brought to nearly unity by advanced superconducting technology^36^. Thus, recalling caveats concerning indistinguishability between distinct near-deterministic sources as discussed in the Introduction, we need to bring non-deterministic nonlinear photon sources into the nearly deterministic regime, that is, increase the source efficiency to at least 0.67 to make optical quantum information processing a reality. The source efficiency is the product of photon-generation efficiency and spectral filtering transmission efficiency. Assuming 90% filter transmission, the photon-generation efficiency has to be greater than 0.75. If we start with a generation efficiency of 0.015 (arbitrarily chosen to be <<1 to suppress multi-pair generation), using the first-order approximation (more rigorous and detailed analysis can be found in refs 22, 23), we need to multiplex at least *N*=0.75/0.015=50 time-bins if the switch circuit has negligible losses. The required number of switches is an integer no less than log_2_*N*+1 (ref. 23), which is 7. Using seven switches, the number of multiplexed time-bins is 2^(7−1)^=64. As this number is larger than the required *N*=50, and the probability of generating more than one pair in 64 time-bins is low since 0.015<1/64, it is possible to achieve the required enhancement. The major challenge is to reduce the losses of switches. Recent development of stress-optic effect-based switches has the potential to bring the switching losses down to the desired level^37^. This type of switch has a 2 μm thick lead zirconate titanate (PZT) film on the top of a very low loss (as low as 0.0005, dB cm^−1^) SiN waveguide^37^. The applied stress has nearly no impact on the loss and thus the switch can be practically lossless if we make the switches and delay lines on the same chip to avoid waveguide-fibre coupling.

The other challenge involved in developing high performance heralded single-photon sources is to have pure heralded photon sources before multiplexing so that the photons after multiplexing are highly indistinguishable. In our current demonstration, the interplay between pump bandwidth and phase matching dictates that purity is maximized with sufficiently narrow filter bandwidths. In the future, we can increase heralded photon purity by either using narrower bandpass filters to appropriately reduce the generated photon bandwidth or employing micro-ring resonators as the nonlinear device^38^.

In conclusion, this demonstration provides a road map for creating near-deterministic heralded single-photon sources using a resource efficient and thus scalable multiplexing scheme. With nearly unity efficiency detectors, 90% transmission filters and low-loss switches, this scheme will ultimately provide a solution for photon sources required for optical quantum computing and simulation.

## Additional information

**How to cite this article:** Xiong, C. *et al*. Active temporal multiplexing of indistinguishable heralded single photons. *Nat. Commun.* 7:10853 doi: 10.1038/ncomms10853 (2016).

## Supplementary Material

Supplementary Information Supplementary Figures 1-2, Supplementary Notes 1-5 and Supplementary References.
