# Supplementary material for: Active temporal multiplexing of indistinguishable heralded single photons
Source: Nat Commun. 2016 Mar 21;7:10853. doi: 10.1038/ncomms10853 (PMC4802115; doi:10.1038/ncomms10853)
Supplement: Supplementary Information — Supplementary Figures 1-2, Supplementary Notes 1-5 and Supplementary References. [file ncomms10853-s1.pdf]

Supplementary Figure 1: Full setup of the experiments

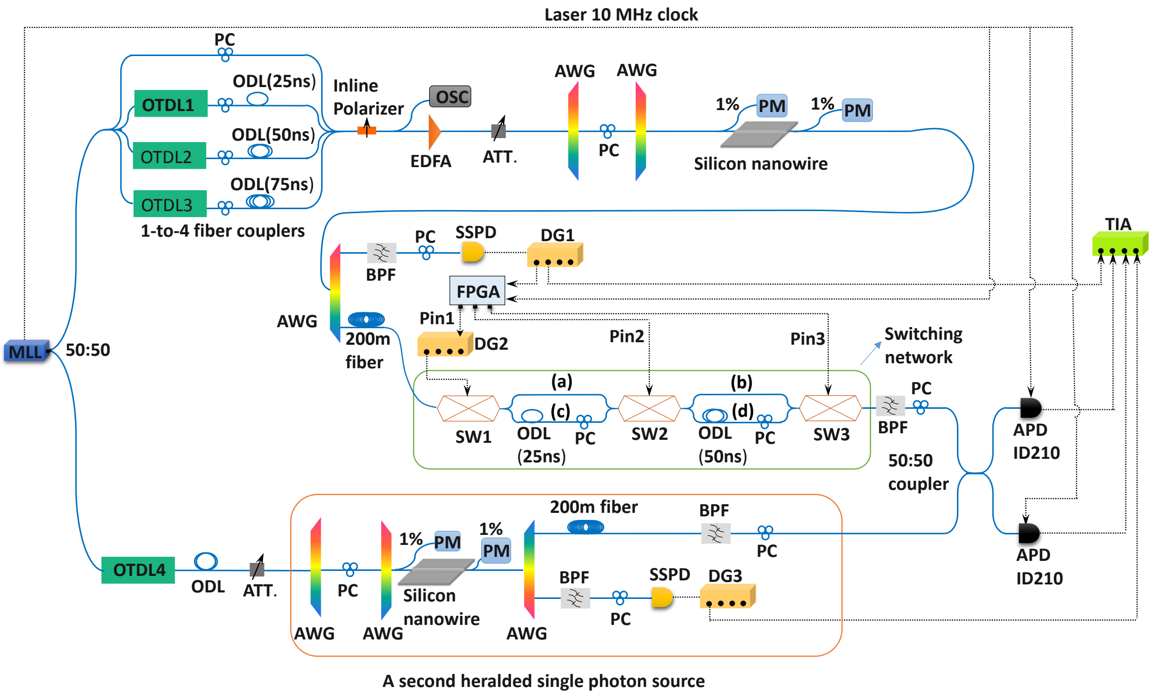

Supplementary Figure 1 | Full setup of the experiments Solid and dashed lines represent optical fibers and electronic cables, respectively. MLL: mode-locked laser, OTDL: optical tunable delay line, ODL: optical delay line, PC: polarization controller, ATT: attenuator, OSC: oscilloscope, AWG: arrayed waveguide grating, PM: power meter, SSPD: superconducting single photon detector, DG: delay generator, FPGA: field-programmable gate array, SW: switch, BPF: bandpass filter, APD: avalanche photon diode, TIA: time interval analyzer.

Supplementary Figure 2: Typical histograms for CAR measurements

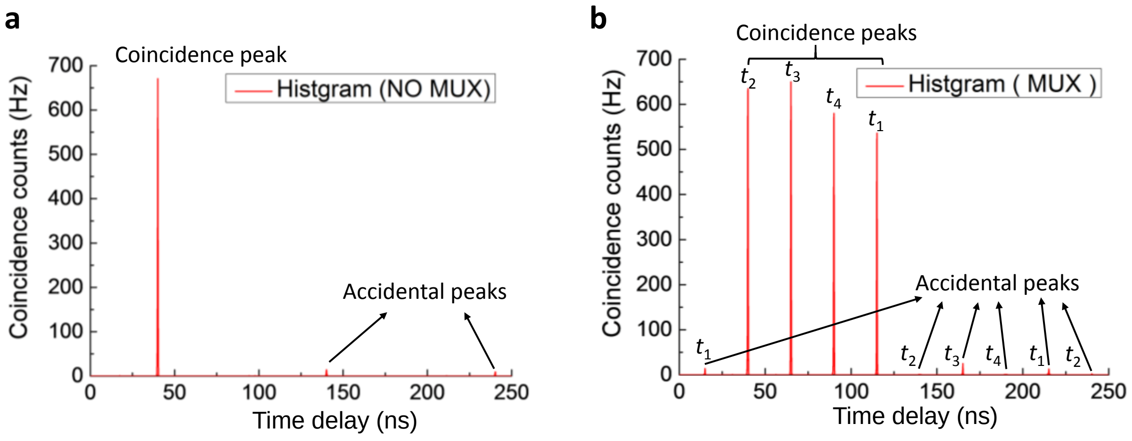

Supplementary Figure 2 | Typical histograms for CAR measurements a, NO MUX: one coincidence peak. **b,** MUX: four coincidence peaks represent photons from four time bins shown in Fig. 1 and two accidental peaks (two nearly disappear because of switching).

Supplementary Note 1: Full setup and explanation of coincidence measurements

The full setup of our experiments is illustrated in Supplementary Figure 1. The top half of the diagram, excluding the 50:50 coupler before the two avalanche photo diode single photon detectors (ID210, Id-Quantique), is for the CAR measurements of the source with four temporal-mode multiplexing (MUX). When doing the measurements for the source without MUX, we connected only one of the four channels of the 1-to-4 fiber couplers (FCs), and removed the switching network. This arrangement allowed the least change to the experimental conditions for the MUX and NO MUX measurements, and thus guaranteed a fair comparison between them. Because the losses of the four channels of the FCs were slightly different, we used one polarization controller (PC) in each channel and an inline polarizer immediately after the FCs to ensure the pulses in the four temporal modes after the inline polarizer had the same intensity. A fast optical sampling oscilloscope was used to monitor the pulse intensities. The CAR measurements for pumping at the 10 MHz clock of only t_1, t_2, t_3 or t_4 (Fig. 1) indicate that all four NO MUX sources have the same performance, as shown by diamonds in Fig. 3a.

The bottom half of Supplementary Figure 1 is a second heralded single photon source based on a silicon nanowire[1] with the same specification to that used in the MUX experiments. This source was pumped at the laser's 10 MHz clock, and provided a reference for the indistinguishability test of the multiplexed photons via the four-fold HOM quantum interference[2].

When we performed the CAR measurements for the NO MUX and MUX photon sources, we took the 50:50 coupler out of the setup and connected the heralded photon

output directly to one ID210 detector. In both cases, the heralded photon events detected by an ID210 triggered by the 10 MHz laser clock were used as the 'start', and the heralding photon events detected by a SSPD were used as the 'stop' for the TIA to construct the histograms: coincidences vs time delay between 'start' and 'stop'. This 'start' and 'stop' arrangement is different from the standard way of using heralding signals as the 'start' and heralded as the 'stop' just for our experimental convenience to adjust the delays, and it does not change the physics. A suitable electronic delay was applied to the heralding detection signals so that the delay in the histogram was always within the 0–250 ns time window. Supplementary Figure 2 shows typical histograms for the NO MUX and MUX sources in the CAR measurements[3]. For the NO MUX source (Supplementary Figure 2a), we sum up all counts in the coincidence peak and the accidental peak closest to the coincidence peak as the coincidences and accidentals, respectively. The time interval between the peaks is the pump laser period. The ratio between them gives the CAR. For the MUX source (Supplementary Figure 2b), depending on in which time bin the heralded photons are generated, they experience different optical delays to be multiplexed to the 10 MHz clock; however the heralding photons are still on the 40 MHz clock, so there are four coincidence peaks (the signature of successful multiplexing). These peaks look slightly different from one another because photons generated in different time bins experience slightly different losses when propagating through different optical paths. Each coincidence peak should have their corresponding accidental peak. Two accidental peaks nearly disappear because the photons that are generated in time bins t_2 and t_4 require logic '1' be applied to switch1 (Fig. 2). This switching operation on switch1 only allows heralded photons to arrive at the detector when there are heralding events, and therefore switches away all other events that may give rise to coincidences in the accidental peak. To calculate the CAR for the MUX source, we sum up the counts in all four coincidence peaks as the coincidences; and multiply the overall counts in the two visible accidental peaks by two as the accidentals. This process is considered to be fair because: first, the sources pumped by each individual temporal mode have been tested to have the same performance; second, corresponding to the loss differences between the different paths, the accidentals for t_3 should be higher than those for t_2, while the accidentals for t_1 should be lower than those for t_4, and the total accidentals for t_3 and t_1 can approximately represent those for t_2 and t_4.

Supplementary Note 2: Clock, optical delay, and switching network loss management

To make this temporal multiplexing experiment successful, we had to synchronize: (I) the 40 MHz clock of the FPGA and the heralding event so that the FPGA could find out which time bin the heralding photons were generated; (II) the switching electronic signals from the FPGA and the heralded photons arriving at the switches so that proper delays could be applied to the photons; and (III) the relative optical delays in the four channels of the FCs and in the switching network.

For synchronization (I), we used a digital delay generator (DG1 in Supplementary Figure 1) between the SSPD heralding detection signal output and the FPGA. By doing

an AND operation between the heralding detection signals and the FPGA clock, and comparing the FPGA output count rate with that on the SSPD software, we could find the correct delay using DG1. Once this was done, we could proceed to synchronization (II).

To simplify synchronization (II), we configured the FPGA such that according to a heralding event, the three binary-digit output was latched until the next heralding event occurred. For example, if a photon pair was generated in time bin t_1, '000' was applied to the switches until the next pair was generated. If the next pair was generated in time bin t_4, the output of the FPGA would change to '110' (Fig. 2). In this way we only need to synchronize the switching signals that control switch1 with the incoming photons, because once photons pass through switch1, they are routed directly through switch2 and switch3 that are always under the correct logic control.

Because the relative delay between heralding and heralded events was the same no matter in which time bin the photon pairs were generated, we only need to do synchronization for one pump temporal mode. As the initial input status to the switches was '0', we had to choose the pump on the clock of either t_2 or t_4 for synchronization because only these two pump modes required a logic '1' input to switch1 and were possible for delay alignment. In the setup shown in Supplementary Figure 1, we only connected the channel of FCs with 25 ns optical delays (i.e. t_2) and applied a constant output '101' from the FPGA to the switches to take a coincidence measurement as the reference. Then we added a digital delay generator (DG2 in Supplementary Figure 1) between the logic output pin1 of the FPGA and switch1. We could continuously tune the delay of the switching signals to switch1 via DG2 and measure the coincidences. By trial and error, the correct delay was found when the measured coincidences matched with the reference measurement. This delay was then double confirmed by coincidence measurements for the other three temporal pump modes.

Note that DG2 only responds when the output logic from pin1 changes from '0' to '1' and keeps switch1 at the "switching" status for 100 ns, so the use of DG2 here is optional as long as we ensure the signals from the FPGA are sent to all three switches before the incoming photons. The advantages of having DG2 for synchronization are that we have a more accurate control to the switching circuit and thus can route some unheralded photons (i.e., pairs are generated but the heralding photons have been lost) to the dumped port.

For synchronization (III), we manually cut optical fibers with the lengths providing delays of approximately 25 ns and 50 ns and spliced the fibers in paths (c) and (d) of the switching network (Supplementary Figure 1); and then used off-the-shelf fiber-integrated optical tunable delay lines (OTDL) with a tuning step of 1 ps in three channels of the FCs for multiplexing the pump to match the delays in the switching network. Compared with directly using OTDL in the switching network, this arrangement minimized the losses of the multiplexed heralded photons.

Supplementary Note 3: Polarization management

Due to the use of non-polarization maintaining components in the switching network, photons with the same polarization at the input generally will have different polarization at the output if they go through different optical paths. Depending on in which time bin

the heralded photons are generated, they go through the combination of optical paths shown in Supplementary Figure 1, t_1: (a)+(b), t_2: (c)+(b), t_3: (a)+(d), and t_4: (c)+(d). We found that adding one PC in each of (c) and (d) was sufficient to maintain the polarization of the photons. For example, the photons from t_1 and t_2 share the path (b), the PC in (c) can always adjust the polarization of photons to be the same to those in (a). Then (c) becomes equal to (a) in terms of polarization. The same rule applies to (d) and (b) and thus photons from all time bins will have the same polarization at the output of the switching network.

Supplementary Note 4: Inferring heralded single photon output probability from coincidences

Because we did not use photon number resolving detectors, we cannot measure the heralded single photon output probability directly, but we can infer it from the coincidences measured by threshold detectors.

As shown in Supplementary Figure 1, both the generated photons and the pump were filtered using AWGs with the same channel bandwidth of 50 GHz. This arrangement makes it appropriate to use the thermal distribution to describe the photon generation statistics[3]. Using the measured coincidences, we calculate the mean number of generated pairs per 100 ns clock period μ, by taking into account the total losses in the heralding and heralded photon arms. Using $P_1=\mu/(1+\mu)^2$ we infer the heralded single photon generation probability per 100 ns clock period. Using $P_1\eta$ with η being the overall photon pair collection efficiency, we calculate the heralded single photon output probability for the plots in Fig. 3b.

Supplementary Note 5: Four-fold HOM interference measurements

The delay management procedure described in Supplementary Note 2 aligns the photon arrival time to an accuracy of 1 ns, which is determined by the time resolution of the coincidence measurement system. The four-fold HOM interference requires the delay alignment at the accuracy of photons’ coherence time which is of the order of the pump pulse width 10 ps. The measurements were taken in the following steps. First, we connected only the channel of the 1-to-4 FCs without optical delay lines as the pump of the MUX source so that photons were always generated in time bin t_1. By varying the delay of OTDL4 in the second source (see Supplementary Figure 1), we adjusted the fine delay via two-fold HOM interference and fixed the delay on OTDL4. Second, we connected in turn only the channel of the 1-to-4 FCs with OTDL1, OTDL2, or OTDL3, to determine and fix the fine delays for them via two-fold measurements. Third, we connected all four channels of the 1-to-4 FCs to obtain the fully multiplexed source and took the two-fold and four-fold HOM interference measurements by varying the fine delay on OTDL4.

As the pump powers for the four-fold HOM interference measurement were relatively high, we attribute the 69% visibility predominantly to multi-pair noise. To obtain this noise information, we performed four-fold coincidence measurements at the

same fine delays set in the raw measurements by disconnecting input to the 50:50 coupler from the MUX and second photon sources, respectively[4]. Because subtracting these four-fold counts from the raw four-fold counts would subtract the noise due to detector dark count twice, we also measured the four-fold coincidences by disconnecting both sources from the 50:50 coupler, and added these dark count induced four-fold counts back to get the net four-fold coincidences. To understand this process, we give an example: we obtained a raw four-fold coincidence count C_{raw} at a particular delay δt. We measured the multi-pair noise contribution to C_{raw} from two sources at delay δt to be C_{n1} and C_{n2}, and the detector dark count contribution to C_{raw} at delay δt to be C_{d}. The corrected net four-fold coincidence is then $C_{\mathrm{raw}} - C_{\mathrm{n1}} - C_{\mathrm{n2}} + C_{\mathrm{d}}$. We did this correction for all delays shown in Fig. 4a and obtained the data for Fig. 4b. This multi-pair noise subtraction yields a $91 \pm 16\%$ visibility.

Supplementary References

1. Zhang, X. *et al.* Enhancing the heralded single-photon rate from a silicon nanowire by time and wavelength division multiplexing pump pulses. *Opt. Lett.* **40,** 2489-2492 (2015).
2. Harada, K. *et al*. Indistinguishable photon pair generation using two independent silicon wire waveguides. *New J. Phys*. **13,** 065005 (2011).
3. Takesue, H. & Shimizu, K. Effects of multiple pairs on visibility measurements of entangled photons generated by spontaneous parametric processes. *Opt. Commun.* **283,** 276-287 (2010).
4. McMillan, A. R. *et al*. Two-photon interference between disparate sources for quantum networking. *Sci. Rep.* **3,** 2032 (2013).
